# Targeting autophagy in astrocytes: a potential for neurodegenerative disease intervention

**DOI:** 10.3389/fncel.2025.1584767

**Published:** 2025-04-28

**Authors:** Maja Potokar, Jernej Jorgačevski

**Affiliations:** ^1^Laboratory of Neuroendocrinology—Molecular Cell Physiology, Institute of Pathophysiology, Faculty of Medicine, University of Ljubljana, Ljubljana, Slovenia; ^2^Celica Biomedical, Ljubljana, Slovenia

**Keywords:** astrocyte, autophagy, mitophagy, cytoskeleton, neurodegeneration, neuroinflammation

## Abstract

Autophagy contributes to cellular homeostasis by regulating the degradation and recycling of damaged organelles and misfolded proteins. In the central nervous system (CNS), impaired autophagy contributes to inflammation, disrupts cellular metabolism, and leads to the accumulation of toxic protein aggregates that accelerate the progression of neurodegenerative diseases. In addition to its role in protein and organelle turnover, autophagy facilitates the elimination of pathogenic bacteria and viruses, whose infections can also lead to neurological diseases and neuroinflammatory processes. Astrocytes, the most abundant glial cells in the CNS, play a crucial role in maintaining neuronal homeostasis by regulating neurotransmitter balance, ion exchange, and metabolic support. During neurodegeneration, they become reactive, actively participating in neuroinflammatory responses by releasing proinflammatory cytokines, activating microglia, and removing toxic aggregates. Cytokine-mediated responses and metabolic changes in astrocytes influence neuronal viability and neurotransmission. Autophagy in astrocytes plays an important role in tuning the astrocyte-dependent activity of neurons under physiological conditions and in pathological activation of astrocytes by disease, injury or pathogenic stimuli. In this review, we highlight the contribution of astrocytes to neurodegeneration from the perspective of changes in their cytoskeleton, the autophagy process in which the cytoskeleton plays a crucial role, and the metabolic support of neurons. The modulation of autophagy at different stages has the potential to serve as an additional therapeutic target in CNS diseases.

## Introduction

**FIGURE 1:**
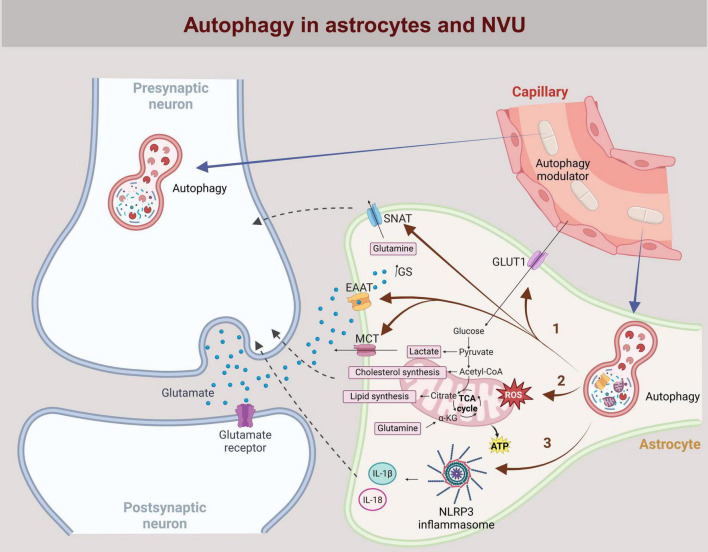
Astrocytic autophagy at the neurovascular interface. The neurovascular unit (NVU) consists of neurons, astrocytes, capillaries lined by endothelial cells, and the perivascular space in between. Neurotransmitters (e.g., glutamate), ions, metabolites (e.g., lactate) and cytokines (e.g., IL-18 and IL-1β) released into the perivascular space by either neurons or astrocytes influence processes in both cell types in a bidirectional manner, including the regulation of autophagy. In astrocytes, autophagy regulates the availability of recycled proteins, influencing the expression of ion channels and transporters (e.g., SNAT, EEAT, MCT, GLUT1) in the plasma membrane. This affects the uptake of key transmitters and metabolites such as glutamate and glucose, as well as the release of glutamine and lactate from astrocytes (pathway 1). Mitophagy, a specific form of autophagy, affects mitochondrial homeostasis and regulates cholesterol and lipid synthesis, as well as the cytoplasmic pool of lactate and glutamine in astrocytes (pathway 2). Additionally, inhibition of autophagy in astrocytes activates the NLRP3 inflammasome, promoting the cleavage of pro-IL-18 and pro-IL-1β into their active forms via caspase1 activation (pathway 3). IL-18 and IL-1β are predominantly released into the extracellular space by exocytosis, modulating inflammation through autocrine and paracrine signaling. Systemically administered autophagy modulators (e.g., rapamycin, simvastatin, everolimus, temsirolimus, BCN4, NMN, berberine, astragalin, resveratrol) permeate through the endothelial cells into the perivascular space and act on neurons and astrocytes. By modulating autophagy in both cell types, these compounds can influence intercellular signaling and neuronal function. GLUT1, Glucose transporter type 1; SNAT, Sodium-coupled neutral amino acid transporter; EEAT, Excitatory amino acid transporter; MCT, Monocarboxylate transporter; BCN4, Beclin 1-derived peptide; NMN, nicotinamide mononucleotide.

Neurodegeneration refers to a pathological condition that primarily affects neurons and occurs in various neurological diseases of the central nervous system (CNS). Although these diseases differ in terms of pathogenetic mechanisms, including different protein aggregates and genetic variations, they all share the characteristic feature of chronic neuroinflammation (Mayne et al., 2020). In addition to peripheral immune cells, glial cells, including microglia, astrocytes, oligodendrocytes, endothelial cells and pericytes, also play a key role in neuroinflammation (Yang and Zhou, 2019). In particular, astrocytes and microglia are closely associated with innate immune responses in the CNS. They detect various cues via surface and cytoplasmic receptors and release proinflammatory cytokines, lipid mediators, chemokines, NO, reactive oxygen species, and secondary messengers to recruit other immune cells and perpetuate the immune response (Zhang et al., 2023). Neuroinflammation is fundamentally a neuroprotective mechanism; however, when chronic or dysregulated, it can contribute to the development or worsening of neurological diseases. Notably, neuroinflammation is not only a consequence of conditions such as Alzheimer’s disease, Parkinson’s disease (PD), multiple sclerosis and Huntington’s disease (HD), but may precede and even contribute to the formation of protein aggregates commonly associated with neurodegeneration (Matthews, 2019; Leng and Edison, 2021; Jia et al., 2022; Tansey et al., 2022). Both the protective and detrimental properties of neuroinflammation are regulated by various complex cellular and molecular processes (Zhang et al., 2023). As recent findings reveal, autophagy in astrocytes is among them (Price et al., 2021). Autophagy in astrocytes could influence various processes in astrocytes, which in turn affect the function of neurons (Figure 1). In neuroinflammation astrocytes undergo phenotypic changes (Price et al., 2021), which are associated with extensive alterations in the cytoskeleton, yet the role of the astrocyte cytoskeleton in autophagy-mediated regulation of neuroinflammation remains unexplored.

Although dysregulated autophagy has been implicated in the pathogenesis of neurodegeneration, acute neuronal injury, aging, brain cancer, infections, and autoimmune conditions, no targeted autophagy-based intervention is currently available for clinical use (Galluzzi et al., 2017). However, several modulators of autophagy have been identified, some of which hold promise for therapeutic intervention in CNS disorders (Galluzzi et al., 2017). Despite this progress, the vast majority of autophagy-related studies have focused on neurons, with astrocytes receiving considerably less attention, at a ratio of approximately 10:1. Astrocytes, which ensheathe capillaries (Abbott et al., 2006), are the first CNS cells to interact with systemically administered drugs crossing the blood-brain barrier (BBB), making them critical mediators of drug effects before reaching neurons (Figure 1). Considering their essential role in CNS homeostasis, including energy metabolism, ion and water balance, and neurotransmitter regulation (Verkhratsky and Nedergaard, 2018), we propose that targeting autophagy in astrocytes is as crucial as in neurons. This review, therefore, explores recent advances in the therapeutic potential of autophagy modulators in astrocytes.

## Astrocytes

Astrocytes, the predominant glial cells in the CNS, play an essential role in supporting neuronal function by regulating neurotransmitter and ion homeostasis, providing metabolic and neurotrophic support to neurons, and also maintaining the BBB (Verkhratsky et al., 2016). Astrocytes are in close contact with the BBB as they are an integral part of the neurovascular unit (NVU), the multifaceted structure in the brain that facilitates blood supply, removal of metabolic waste and protection from potentially harmful peripheral material (McConnell et al., 2017). The NVU consists of arteries that supply blood, endothelial cells, the perivascular space that supports glymphatic waste clearance, astrocytes and neurons (Sun et al., 2021). On the one hand, astrocytes are an integral third component of the synapses formed by neurons; on the other hand, they enwrap ∼99% of the vessel on the abluminal side (Filosa et al., 2016). Within the NVU astrocytes respond promptly to changes in synaptic activity and neuronal metabolism, to help regulate cerebral blood flow (McConnell et al., 2017). The proper functioning of astrocytes requires constant remodeling of astrocytic processes, which depends on changes in cytoskeleton organization and distribution, as well as modifications in water transport across the astrocyte’s plasma membrane. The abundant expression of the water channel aquaporin-4 on astrocyte endfeet is critical for the perivascular clearance of extracellular metabolic waste from neurons into the glymphatic system (Iliff et al., 2012). The capacity of the glymphatic system also depends on the intracellular waste-removal system, specifically the autophagy pathway. When autophagy of neuronal cells is impaired, the build-up of potentially harmful substrates occurs in the perivascular space (Natale et al., 2021). When autophagy is impaired in astrocytes, this likely contributes to the accumulation of potentially toxic substrates and to impaired metabolic and signaling support to neurons (Figure 1). To alleviate the burden of accumulating potentially toxic waste products, numerous strategies are being investigated, including the direct and indirect targeting of cytoskeleton-dependent processes, such as autophagy. Astrocytes are among the first cells to come into contact with molecules attempting to enter the brain (Kadry et al., 2020; Jorgacevski and Potokar, 2023). This also applies to drug delivery, an intensively researched area that aims to better control cellular processes in neuronal cells. Targeting the neurodegeneration-related autophagy process in astrocytes could play a significant role in the development and progression of neurodegeneration and neuroinflammation.

In the CNS pathological stimuli lead to an inflammatory response, mediated first by microglia and by infiltrating peripheral immune cells and then by astrocytes. Astrocytes adapt to pathologic conditions by undergoing morphologic and molecular changes, a process known as reactive astrogliosis (Baldwin et al., 2023). These alterations of astrocytes lead to the loss of certain homeostatic functions while simultaneously acquiring either protective or harmful roles (Escartin et al., 2021). Reactive astrocytes play a crucial role in neurodegeneration also by contributing to chronic neuroinflammation (Escartin et al., 2021; Jorgacevski and Potokar, 2023). It is remarkable that, despite the abundance of astrocytes and their critical roles in CNS homeostasis, processes governing reactive astrogliosis remain largely unknown. It is known, however, that morphological adaptations accompanying reactive astrogliosis are associated with differential expression of intermediate filaments (IFs) while dysregulated autophagy both contributes to and results from astrocyte reactivity (Chandrasekaran et al., 2021).

In addition to IFs, such as vimentin and glial fibrillary acidic protein (GFAP), microtubules (MT) and the actin filaments (AF) also play an important role in regulating astrocyte morphology and structural changes during reactive astrogliosis (Potokar et al., 2020). A key protein that links all three major filament systems and facilitates their binding to intracellular targets is the cytolinker protein plectin (PLEC), which has been shown to affect cellular and organelle morphology in fibroblasts (Winter et al., 2015). PLEC was discovered in the C6 cell line of rat glioma and is primarily an IF cytolinker protein. Recently, it has been suggested to modulate the morphological adaptations of reactive astrocytes (Potokar and Jorgacevski, 2021). Astrocytes express PLEC in copious amounts and its expression is further increased in reactive astrocytes, making it a promising candidate as a marker for reactive astrogliosis in pathological conditions (Potokar et al., 2020). Isolated primary and immortalized astrocytes mainly express three PLEC isoforms: P1c, P1e, and P1g, among which P1c is the predominant isoform (Zugec et al., 2024). In PLEC-null immortalized astrocytes defects in collective migration and volume regulation have been detected (Zugec et al., 2024). The exact role of PLEC in reactive astrocytes at the cellular level and in the context of specific CNS functions, remains to be investigated. In addition to changes in PLEC expression, upregulation of IFs, particularly GFAP, is a hallmark of reactive astrocytes. Vimentin, nestin and synemin are also re-expressed, after their postnatal downregulation, and their intracellular arrangement alters in different neurological conditions (Potokar et al., 2020). The reorganization of IFs in astrocytes not only alters cellular morphology but also regulates the positioning and mobility of vesicles and organelles (Potokar et al., 2010; Zugec et al., 2024). Mammalian target of rapamycin (mTOR) activation has been shown to trigger endolysosome remodeling, manifested as alkalinization of LAMP1-positive compartments, and promote the endolysosomal exocytosis (Leng et al., 2024), but the role of the cytoskeleton in this mechanism has not been investigated. More attention needs to be paid to the release of cytokines by reactive astrocytes, as together with gliotransmitters they profoundly influence intercellular communication, BBB permeability and neuroinflammation (Osipova et al., 2018). Autophagy is closely linked with inflammatory and immune responses, as it regulates cytokine production (Jiang et al., 2019), while cytokine secretion in reactive astrocytes triggers further release of cytokines (Chandrasekaran et al., 2021). To date, the involvement of reactivated expression of IFs in the transport of cytokines and autophagic compartments in astrocytes has not been addressed. In other models of injury, for example in acute lung injury, vimentin has been shown to directly interact with NLRP3 inflammasome, triggering its assembly and activation (dos Santos et al., 2015). And embryonic kidney cells vimentin has been reported to affect localization of autophagosomes and their fusion with lysosomes (Biskou et al., 2019). Since vimentin expression is upregulated in reactive astrocytes, it is plausible that vimentin, along with other IFs and PLEC, may influence autophagy at multiple stages, including the translocation of phagophores and autophagosomes within the cytoplasm. This notion is supported by the observation in retinal pigment epithelial cells that the absence of PLEC results in the collapse of keratin filaments, which attenuates the fusion of autophagosomes and lysosomes (Son et al., 2022). Considering the recent findings that PLEC deficiency in astrocytes induces the bundling and redistribution of AF and vimentin filaments in perinuclear and subplasmalemmal regions, it can be assumed that cytoskeletal filaments and PLEC are important for the progression of autophagy in astrocytes as well. Although autophagy has attracted considerable attention as a target for the development of novel therapeutics (Galluzzi et al., 2017), there are currently no clinically available drugs that effectively modulate autophagy in astrocytes.

## Autophagy

**FIGURE 2:**
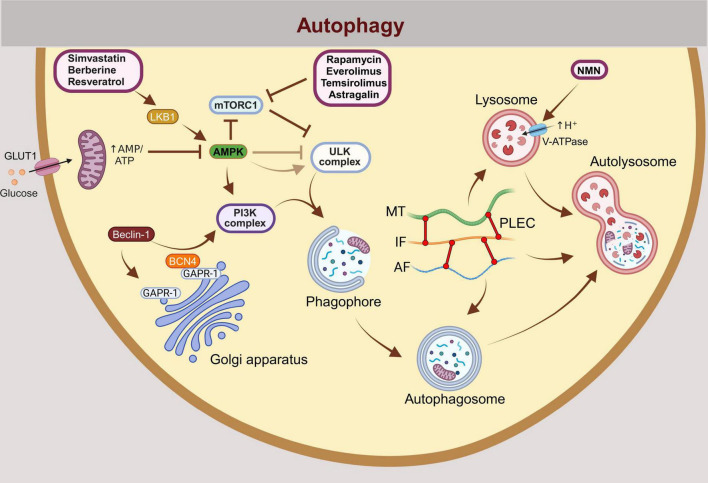
Autophagy, including mitophagy, is a multistep process regulated by numerous proteins, many of which represent potential targets for the treatment of neurodegeneration and neuroinflammation. The initiation of autophagy is regulated by the activation or inhibition of mTORC1, ULK1, and the PI3K complexes. Under conditions of energy stress, AMPK is activated by an increased AMP:ATP and ADP:ATP ratio, promoting autophagy. Autophagy is initiated when mTORC1 inhibition relieves its suppression of the ULK1 complex. Physiologically, this occurs during starvation (e.g., glucose deprivation), which induces autophagy through the activation of AMPK. The prevailing model suggests that AMPK phosphorylates and activates ULK1 (UNC-51 like kinase 1), initiating autophagy. However, new evidence indicates that glucose deprivation may suppress ULK1 signaling despite AMPK activation. When autophagy is induced, a phagophore membrane structure forms and elongates until it fully closes to form autophagosome. The autophagosome subsequently fuses with a lysosome to form an autolysosome, where hydrolytic enzymes degrade the autophagic cargo in an acidic environment. External autophagy modulators that have the potential to treat neurodegenerative diseases by inducing autophagy are shown in the scheme: Rapamycin, Everolimus, Temsirolimus (mTORC1 inhibitors), Simvastatin (activator of the LBK1-AMPK-mTOR signaling pathway), Berberine, Resveratrol (activators of AMPK and class III PI3K complex), NMN (increases acidification of lysososme). BCN4 prevents Beclin-1 from binding to GAPR-1, maintaining its availability for PI3K complex-mediated autophagy initiation. GLUT1, Glucose transporter type 1; mTORC1, Mammalian target of rapamycin complex 1; ULK1, Unc-51-like autophagy activating kinase 1; PI3K, The class 3 phosphoinositide 3-kinase (PI3K); AMPK, 5′-adenosine monophosphate-activated protein kinase; NMN, nicotinamide mononucleotide; BCN4, Autophagy-inducing peptide derived from the evolutionarily conserved domain of Beclin 1; GAPR-1, Golgi-associated plant pathogenesis related protein 1 (GAPR-1) membrane-binding protein that strongly associates with the cytosolic leaflet of Golgi membranes.

Macroautophagy is a type of autophagic process in which substrates are sequestered within cytosolic double-membrane vesicles termed autophagosomes. Macroautophagy, a primary degradation pathway, which is in this manuscript referred to as autophagy, serves to recycle the cytoplasm, generate energy, and remove damaged organelles and proteins to prevent their accumulation under stress conditions. Material destined for degradation by autophagy is gradually enclosed by a double membrane forming the phagophore, which matures into an autophagosome and eventually fuses with the lysosome, where macromolecules are then degraded (Figure 2; ***Feng et al., 2014)***. The regulation of autophagy is complex, with the mTOR (mammalian target of rapamycin)-dependent pathway playing a central role. In addition, mTOR-independent pathways, including AMPK, PI3K, Ras-MAPK, p53, PTEN, and endoplasmic reticulum stress, also contribute to its regulation ***(Perez-Alvarez et al., 2018)***.

### Autophagy in astrocytes

Research of autophagy in astrocytes and its significance for neurological disorders has been expanded in recent years. Of particular interest is the question of whether autophagy in astrocytes has a cytoprotective or cytotoxic effect on the surrounding cells, especially the neurons. Astrocytes exhibit a high rate of basal autophagy, suggesting that their autophagy process is crucial for their supportive role in the brain (Gabryel et al., 2017). Central autophagy-regulating genes in neurons and astrocytes are important for normal brain development and function (Hara et al., 2006). Autophagy in astrocytes can be increased even further, as has been demonstrated under starvation conditions and during viral infections (Tavcar Verdev et al., 2022). It has also been reported that upregulation of autophagy flux in astrocytes can be neuroprotective after stroke, as induction of astrocyte autophagy flux *in vitro* increased neuronal viability and decreased neuronal apoptosis (Liu et al., 2018). Autophagy in astrocytes also plays a crucial role in regulating inflammation following viral infections, which has been recently reviewed (Jorgacevski and Potokar, 2023).

Autophagic flux is also controlled by the cytoskeleton, yet relatively little is known about the role of specific elements of the cytoskeleton (Monastyrska et al., 2009). Studies addressing the role of MT discovered that they regulate trafficking of autophagosomes to fuse with lysosomes and form autolysosomes (Ravikumar et al., 2008) and when this step is blocked, the autophagosomes enlarge (Webb et al., 2004). For example, in the brains of AD patients, pathogenic tau, which is typically degraded by macroautophagy, causes MT degradation. This disruption impairs autophagosome-lysosome fusion, leading to a massive accumulation of immature autophagic structures in axons (Caballero et al., 2021). In other cell types, MT also facilitate the formation of autophagosomes and disruption of MT reduces the fusion of autophagosomes with late endosomes/lysosomes, leading to a delay in autophagy, as demonstrated in CHO, HeLa cells and hepatocytes (Fass et al., 2006; Kochl et al., 2006). AF dynamics also play an important role in autophagy. The branched AF network is critical for the biogenesis of autophagosomes from the endoplasmic reticulum membrane and for the transport of ubiquitinated cargo, including mitochondria and protein aggregates, to the growing phagophore (Kast and Dominguez, 2017). In the CNS, the role of cytoskeletal filaments in autophagy, especially IFs, is beginning to be explored but it has not yet been investigated in astrocytes. In contrast, in neurons, IF peripherin has been demonstrated to fine-tune lysosomal biogenesis, positioning and functions of lysosomes, thus affecting both the endocytic and autophagic pathways (Romano et al., 2025). Studies in other cell types, such as human embryonic kidney cells, show that vimentin, AF and MT are important regulators of autophagosomal vesicle positioning (Biskou et al., 2019). For example, depolymerization of vimentin filaments led to vimentin aggregation and thus to relocalization of autophagosomes and lysosomes to a juxtanuclear location (Biskou et al., 2019). The final fusion of autophagosomes in the autophagic pathway occurs with lysosomes, which are degradation foci for endocytic and autophagic components (Futerman and van Meer, 2004). The importance of the cytoskeleton for the transport of vesicles and organelles in reactive astrocytes has already been investigated in several studies, but not yet for autophagic compartments. For example, the transport of lysosomal vesicles, recycling, endocytotic and exocytotic vesicles in astrocytes is critically affected by the reorganization of MT, AF and IF networks (Potokar et al., 2008). Similarly, the IF-associated cytolinker protein plectin influences directionality of lysosomal and mitochondrial translocation in neurons (Valencia et al., 2021). Given that the transport of lysosomes, the organelles with which autophagosomes eventually fuse to complete autophagosomal degradation, is critically dependent on IFs and PLEC, it should be assumed that they also play a crucial role in several phases of autophagy in astrocytes, including mitophagy.

### Mitophagy in astrocytes

**FIGURE 3:**
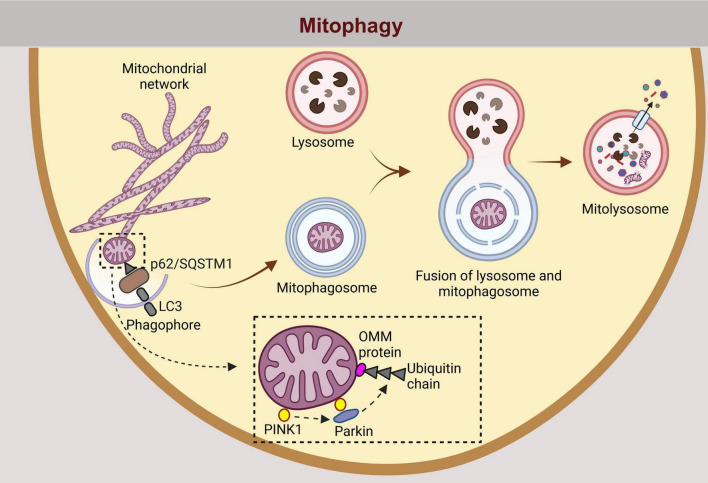
Similarly, mitophagy, the selective degradation of damaged or dysfunctional mitochondria, follows an analogous pathway. Dysfunctional mitochondria are tagged for degradation by autophagy adaptors such as p62/SQSTM1, which contains binding domains for ubiquitin and LC3. The process is facilitated by the ubiquitination of outer mitochondrial membrane OMM proteins by parkin, following recruitment and phosphorylation by PINK1. p62/Sequestosome-1 (SQSTM1), a selective autophagy receptor for degradation of ubiquitinated substrates; LC3, Microtubule-associated protein 1 light chain 3 beta; PINK1, Phosphatase and Tensin homolog (PTEN)-induced putative kinase 1; OMM, outer mitochondrial membrane.

Mitophagy is a specialized process of macroautophagy in which dysfunctional (damaged and depolarized) mitochondria are degraded (Figure 3), contributing to mitochondrial homeostasis in cells (Zimmermann et al., 2024). Damaged and depolarized mitochondria are degraded by mitophagy (Figure 3), a specialized process of macroautophagy (Picca et al., 2023). Depending on the physiological needs and roles of cells in different tissues, three types of mitophagy can be distinguished: basal mitophagy, stress-induced mitophagy, and programmed mitophagy (Picca et al., 2023). Mitophagy facilitates the removal of damaged mitochondria and thus promotes the formation of new, functional mitochondria, which is important for maintaining mitochondrial health and efficiency. Although the distinction between basal and induced mitophagy remains unclear, the principle of mitophagy essentially follows autophagy, starting with the encapsulation of mitochondria in a double-membrane vesicle, followed by fusion with a lysosome and gradual degradation to recycle the basic building blocks delivered back to the cytoplasm (Zimmermann et al., 2024). Dysfunctional or damaged mitochondria lose the mitochondrial membrane potential, which is a trigger for the recruitment of phosphatase and tensin homolog (PTEN)-induced kinase 1 (PINK1) to the outer mitochondrial membrane. PINK1 is a mitochondrial signaling kinase that fulfills many functions, including transport, fusion and fission of the mitochondrial network, and mitophagy (Ziviani et al.). PINK1 is a mitochondrial damage sensor that recruits and phosphorylates Parkin, which increases E3 ligase activity and leads to ubiquitylation of outer mitochondrial membrane proteins and initiation of autophagy (Figure 2). The connection between ubiquitinated mitochondria and the autophagic membrane requires adaptor proteins, as ubiquitin chains do not bind directly to ATG8 proteins or the autophagic membrane itself (Wang et al., 2023). These adaptor proteins, primarily sequestosome 1 (P62/SQSTM1), have both a ubiquitin-binding domain that recognizes ubiquitin-tagged mitochondria and a domain that binds ATG8/LC3 (Matsumoto et al., 2011). LC3 are MT-associated proteins 1A/1B light chain 3B, which are required for the formation of the phagophore, its elongation and the maturation of the autophagosome in macroautophagy and mitophagy. Following the formation, autophagosome then fuses with the lysosome, which results in degradation of autophagosome cargo (Wang et al., 2023). While the involvement of IFs in astrocytic autophagy has not been studied yet, vimentin suppression in lung fibroblasts has been shown to reduce autophagy and heighten mitochondrial oxidative stress and inflammation. On the other hand, overexpression of vimentin partially protected lung fibroblasts from oxidative stress-induced damage and autophagy dysfunction (Pan et al., 2021). Recent results suggest that vimentin may disrupt the inflammatory cascade by activating the mitochondrial autophagy pathway in early-stage lung fibroblasts (Pan et al., 2021; Picca et al., 2023). In astrocytes, vimentin is densest around the nucleus, forming a so-called vimentin cage, a structure that is highly dependent on the cytolinking function of PLEC (Wiche and Winter, 2011; Zugec et al., 2024). Given its partial overlap with perinuclear mitochondria, the vimentin cage may influence mitophagy in astrocytes. Similarly, the actin cage in HEK293 cells has been linked to mitophagy by encasing dysfunctional mitochondria so that they cannot fuse with the rest of the mitochondrial network. The molecular motor MYO6, which moves along the AF, tethers endosomes to the AF to facilitate the maturation of mitophagosomes and the fusion of autophagosomes and lysosomes (Kruppa et al., 2018). Mitophagy in astrocytes remains largely unexplored, though it has been shown it is essential for optic nerve health (Yazdankhah et al., 2023).

### Dysregulation of autophagy and mitophagy in astrocytes and neurodegeneration

Increasing evidence suggests that astrocytes play a key role in the initiation and development of neurodegenerative disorders (Deng et al., 2024). In neurodegenerative disorders there is also a widespread dysfunction of autophagic processes in the brain, in particular in neurons, but also in glial cells, resulting in impaired clearance of misfolded proteins and thus their accumulation, e.g. amyloid beta peptide (Aβ) in AD, α-Synuclein (α-Syn) in PD, huntingtin in HD resulting in neuroinflammation (Guo et al., 2018). Neuroinflammation and autophagy are interconnected in a bidirectional relationship, where inflammation can influence autophagy, and autophagy, in turn, can modulate inflammatory processes (Kim et al., 2024). Reactive astrocytes surrounding Aβ plaques facilitate Aβ clearance by uptake and subsequent predominant autophagic degradation of Aβ and by secretion of Aβ-degrading proteases into the extracellular environment (Frost and Li, 2017). Prior to degradation, internalized Aβ influences the expression of autophagy genes and thus modulates autophagic flux in astrocytes (Kim et al., 2024). In PD astrocytes are important for the endocytosis of extracellular α- Syn released by neurons (Tsunemi et al., 2020). And similar to Aβ, also α-Syn is in astrocytes primarily degraded by the autophagy-lysosome system, however the clearance of α-Syn is delayed in senescent cells because of impaired autophagic activity (Hong et al., 2024). In addition to the removal of toxic aggregates, autophagy in astrocytes can regulate the release of pro-inflammatory cytokines and the activation of microglia. Any dysregulation of autophagy, either its overactivation or inhibition, may exacerbate neuroinflammation and contribute to neurodegeneration. The contribution of autophagy to neuroinflammation has been extensively reviewed elsewhere (Gan et al., 2024). In brief, autophagy can promote inflammation, regulate the expression of inflammatory factors and act as an effector of inflammation that is regulated by cytokines, i.e., TNF-α, interferon (IFN)-γ, interleukin-1β (IL-1β), IL18, IL-4, IL-10 and IL-17 (Gan et al., 2024). Pharmacological and genetic modulation of astrocytic autophagy pathways may strengthen the detoxification machinery of astrocytes under AD stress and could be a therapeutic strategy to ameliorate AD pathology (Kim et al., 2024).

Mitochondrial dysfunction in reactive astrogliosis also plays a crucial role in the progression of CNS pathologies and is thus an important target for therapeutic interventions to modulate astrogliosis and alleviate neurodegenerative conditions. Mitochondrial defects that impair their dynamics, transport and exchange have been associated with aging and various neurodegenerative diseases, including the two most common, AD and PD (Martinez-Vicente, 2017). Impaired mitophagy has been observed in AD, PD, HD and amyotrophic lateral sclerosis, as detailed in other reviews (Wang et al., 2023). The hyperphosphorylated tau protein (pTau) and the toxic Aβ, which is formed by cleavage from the amyloid precursor protein (APP), accumulate in the AD brain (De Strooper and Karran, 2016). APP is located in the mitochondria and Aβ can be formed either in the mitochondria or imported into the mitochondria. Previously, it was thought that only neurons express enzymes for the production of Aβ peptide, but reactive astrocytes also have elevated levels of the three necessary components for Aβ production, which are APP, β-secretase (BACE1) and γ-secretase (Frost and Li, 2017). Neuroinflammation even promotes astrocytic Aβ production (Frost and Li, 2017). Soluble Aβ has a variety of physiological functions, including modulating synaptic function, facilitating neuronal growth and survival, protecting against oxidative stress, and monitoring against neuroactive compounds, toxins, and pathogens (Bishop and Robinson, 2004). APP and Aβ influence mitochondrial function, and mitochondrial function alters Aβ production from APP (Wilkins, 2023). Mitophagy appears to play an essential role in the clearance of APP that forms amyloid plaques (Vaillant-Beuchot et al., 2021). How these interactions contribute to the AD pathology and progression remains unknown, but impaired mitochondrial homeostasis plays a pivotal role in AD pathogenesis (Wilkins, 2023). The degree of cognitive dysfunction in AD has been linked to the extent of Aβ accumulation in mitochondria and mitochondrial abnormalities (Dragicevic et al., 2010). Mitochondrial dysfunction is also an important factor in PD, sporadic and familial alike (Exner et al., 2012). PINK1 and Parkin are key molecules in the regulation of mitophagy and are mutated in autosomal-recessive juvenile parkinsonism (Nguyen et al., 2016). Mutations in the parkin gene PARK2 are the most common genetic risk factors for the early onset of PD (Marder et al., 2010). PD also correlates with mutations in α-Synuclein (α-syn), leucine-rich repeat kinase 2 (LRRK2), protein DJ-1 (DJ-1), F-Box protein 7 (Fbxo7) and vacuolar protein sorting 35 (VPS35), as reviewed in (Wang et al., 2023). Ineffective mitophagy in animal models of Parkinson’s disease has been associated with inflammatory responses, accumulation of mitochondria in neurons and death of dopaminergic neurons (Liu et al., 2019). The AMPK-dependent ULK1 signaling pathway is also important for mitophagy, as activated ULK1 rapidly phosphorylates Parkin on impaired mitochondria (Hung et al., 2021). Dysfunctional ULK1 and downregulated BNIP3L in AD-affected brain lead to impaired mitophagy, which manifests in impaired initiation steps of the mitophagy process (reduced recruitment of activated LC3 to mitochondria) and an accumulation of structurally and functionally damaged mitochondria (reduced size, disorganized cristae and low ATP production) (Fang et al., 2019). Thus, activation of the AMPK/mTOR/ULK1 signaling pathway may be beneficial in AD-related pathologies by promoting autophagy, as demonstrated in *in vivo* and *in vitro* AD models (Wang and Jia, 2023). The mitophagy receptor BNIP3L/Nix located in the outer mitochondrial membrane, is an emerging target to enhance mitophagy for the treatment of PD, as BNIP3L/Nix appears to compensate for the loss-of-function mutation of the PRKN/PARK2 and restores mitophagy. In contrast to neurons, the involvement of ULK1 and BNIP3L/Nix in mitophagy in astrocytes has not yet been systematically investigated. One of the few studies on mitophagy in astrocytes focuses on mutations in the *POLG* gene, which encodes the catalytic subunit of DNA polymerase γ that is critical for mitochondrial DNA (mtDNA) repair and replication. It was proposed that *POLG* mutations may play a role in some neurodegenerative diseases, including frontotemporal dementia and dementia with Lewy bodies (Borgione et al., 2023).

The role of the cytoskeleton in mitophagy of reactive astrocytes is basically unknown. However, the astrocytic IF network is expected to play a role in the regulation of mitochondrial homeostasis, including mitophagy. The major IF cytolinker PLEC has already been shown crucial for mitochondrial dynamics, as demonstrated in skeletal muscle cells, where it regulates mitochondrial fusion and fission (Winter et al., 2015). Importance of PLEC for mitochondrial dynamics has also been shown in neurons, where those lacking the P1c isoform showed impaired MT dynamics, excessive tau association, and impaired transport of mitochondria and vesicles (Valencia et al., 2021). As P1c is the prevalent PLEC isoform in astrocytes (Zugec et al., 2024), similar disturbances in mitochondrial dynamics and MT organization are to be expected during reactive astrogliosis. In addition, the involvement of PLEC in MT-driven mitophagy has been observed in retinal pigment epithelial cells, where it connects mitochondria to keratin 8 and triggers protective fusion-associated mitophagy (Baek et al., 2021). Although the link between IFs, PLEC and mitophagy in astrocytes is still unexplored, their established roles in other cell types suggest that they may be relevant for astrocyte adaptation in CNS pathologies.

The progression of neurodegenerative diseases may differ between males and females. In the case of AD, women are 2–3 times more likely to be affected after the age of 65 develop than men (Aggarwal and Mielke, 2023). On the other hand, the prevalence of PD is 1.5–2 times higher in men than in women, suggesting that there may be gender specific factors influencing the progression of the disease (Iwaki et al., 2021). Studies looking at the effect of estrogen on autophagy, demonstrated the association between estrogen treatment and the induction of autophagy in several cancers, as described in detail (Xiang et al., 2019). As discussed in the paper, estrogen affects the signaling network involved in the expression of autophagy-related genes and regulates the induction of autophagy or the reduction of overstimulated autophagy. The fact that astrocytes express estrogen receptor beta, which is important for cognition, suggests that estrogen-modulated autophagy in astrocytes may indirectly influence cognition in neurodegenerative diseases (Itoh et al., 2023).

Future research should aim to investigate the molecular pathways that govern autophagic processes in response to CNS injury and disease.

### Modulators of autophagy in astrocytes

Numerous modulators of autophagy have already been discovered (Figure 2). However, in order to exert their effect in the brain, they must pass through the BBB. The impermeability of the BBB prevents almost 100% of large-molecule neurotherapeutics and more than 98% of all small-molecule drugs from entering the brain (Pardridge, 2005). Drug molecules can pass through the BBB via several pathways, including paracellular and transcellular diffusion, receptor-, cell-, transporter- and adsorption mediated transcytosis (Figure 1; Tajes et al., 2014). The endothelial cells of the brain form the main interface with the capillaries, but the tight seal of the BBB as a whole is achieved in concert with other cells that form the NVU, astrocytes, pericytes, and neurons (Tajes et al., 2014). Tightly interconnected capillary endothelial cells are encircled by astrocyte endfeet over the continuous basal lamina and help maintain a highly restrictive BBB (Abbott et al., 2006). Research on autophagy modulators in the brain, including their entry and effect on specific autophagy stages, is mostly limited to neurons. However, these autophagy modulators likely exert similar effects in astrocytes.

Pharmacologically available autophagy modulators act at different stages of autophagy, such as the nucleation, elongation, fusion or degradation phase (Figure 2). The enhancement of autophagy can be used to improve neurodegenerative diseases, as an impairment of autophagy has been demonstrated in samples from patients with various forms of neurodegeneration (Palmer et al., 2025). Autophagy inducers also act on mitophagy by promoting the degradation of damaged mitochondria from the cytoplasm. Agents that enhance autophagy or processes associated with autophagy may be beneficial, as has been shown in animal models of neurodegeneration. In neurodegenerative disorders autophagy is inhibited and its activation can lead to symptom relief (Hara et al., 2006). The activation of autophagy in mammalian cells can be achieved by several modulators. Autophagy and mitophagy activators that permeate the BBB are summarized in Table 1. One of the best known autophagy inducers is rapamycin, which targets the serine/threonine protein kinase mTOR, which controls cell growth, proliferation, and survival (Ballou and Lin, 2008). In many human cancers, mTOR signaling is upregulated, as it is in AD patients, and this upregulation leads to a decrease in autophagy (Li et al., 2005; Ballou and Lin, 2008; Bhutia et al., 2013; Francois et al., 2014). Rapamycin and its analogs are highly selective inhibitors of mTOR and are already used clinically for the treatment of cancer, but an important aspect should be considered in the treatment of AD. Based on preclinical data on the use of rapamycin in the treatment of AD, it is strongly recommended that rapamycin be used with great caution, as in the later stages of AD, the brain’s lysosomal system is severely damaged and treatment with rapamycin is likely to exacerbate this damage. Hence, rapamycin should be considered in earlier stages of AD to alleviate further neuropathology (Carosi and Sargeant, 2019). Systemic or intracerebral administration of rapamycin also activated autophagy in mouse models of PD (Liu et al., 2023). Due to the limited uptake of rapamycin into the body, the development of so-called rapalogs is under way, including for example temsirolimus, everolimus and ridaforolimus (Frake et al., 2015). Temsirolimus is currently used in the treatment of renal cell carcinoma, glioblastoma (Wang et al., 2011). Everolimus is a drug used to treat various types of cancer, including glioblastoma (Zureick et al., 2019). Everolimus-induced mTOR inhibition has been shown to reduce human APP/Aβ and tau levels and improve cognitive function and the depression-like phenotype in the 3 × Tg-AD mouse model of AD (Cassano et al., 2019). In mice expressing HD-associated variants of human huntingtin rapamycin temsirolimus and everolimus exerted neuroprotective effects, as determined histologically and in behavioral tests (Ravikumar et al., 2004). Simvastatin has been shown to lower Aβ concentrations in yeast and reduce cerebral Aβ42 and Aβ40 concentrations in cerebrospinal fluid and brain homogenate (Fassbender et al., 2001; Dhakal et al., 2019). In addition to drugs originally discovered for the treatment of various diseases, new peptides are also being synthesized, such as BCN4, which is derived from the Beclin 1 protein. The protein Beclin 1 is the smallest and most important component of the class III phosphatidylinositol 3-kinase (PI3K) complex and plays a central role in the autophagy process by regulating the early (i.e., autophagosome formation) and late (i.e., autolysosome formation) steps of autophagy (McKnight and Zhenyu, 2013). Beclin1, a key protein for the formation of autophagosomes, is reduced in the brains of AD patients (Crews et al., 2010). In tuning autophagy, Beclin-1 binds to Golgi-associated plant pathogenesis-related protein 1 (GAPR-1), which is a negative regulator of autophagy (Glover et al., 2017). Sequestration of cytosolic Beclin 1 by GAPR-1 restricts the ability of Beclin-1 to induce autophagy. Like TAT-BENC1, BCN4 competes with Beclin 1 for binding to the negative regulator of autophagy, GAPR-1 (Amin et al., 2024). Both peptides, BCN4 and TAT-BENC1, are derived from the Beclin 1 subunit of the class III PI3K complex, but unlike TAT-BENC1, BCN4 crosses the intact BBB (Amin et al., 2024). Nicotinamide mononucleotide (NMN), which increases acidification of lysosomes, has recently been reported to exert anti-AD effects by activating autophagy in AD mice (Ma et al., 2024). As shown in the same study, NMN enhanced autophagy at the cellular level in rat pheochromocytoma cells (PC12 cells), which promoted the degradation of neurofibrillary tangles consisting of tau protein. Experiments on NMN-3xTgAD mice have shown improved mitochondrial dynamics and reduced Aβ and p-Tau pathologies, which aligned with better cognitive performance. In addition, NMN-treated mice exhibited increased expression of Sirtuin 1, a protein known to attenuate mitochondrial oxidative stress (Liu et al., 2013). It would be interesting to investigate whether a similar effect is also observed with protein aggregates associated with other neurodegenerative diseases. The highly acidic luminal pH of lysosomes is central to their function and has been shown to play a central role in the pathogenesis of various diseases, including neurodegenerative disorders, metabolic diseases, infections, and cancer. Therefore, several autophagy modulators target this step, offering potential for therapeutic intervention (Lo and Zeng, 2023). While some autophagy modulators are well established, others are less well studied, such as BCN4 and naturally occurring biologically active compounds (NMN, astragalin, berberine, resveratrol). For clinical application it needs to be clarified which modulators are more specific and cause fewer adverse effects.

**TABLE 1:** Blood-brain barrier permeable autophagy activators.

Autophagy modulator	Action	Effect on autophagy	References
**Activators of autophagy tested in astrocytes**						
Rapamycin (ingredient: sirolimus, brand name: rapamune)	Inhibition of mTORC1	Increase in the number of autophagic compartments, increase of autophagy flux	(Viscomi et al., 2012; Tavcar Verdev et al., 2022)
Simvastatin	Activation of the LBK1-AMPK-mTOR signaling pathway	Increase of autophagy flux	(Dhakal et al., 2019)
**Activators of autophagy not tested in astrocytes**						
Everolimus (ingredient: everolimus, brand names: afinitor, zortress, torpenz, afinitor disperz)	Inhibition of mTORC1	Increase of autophagy flux	(Zureick et al., 2019)
Temsirolimus (ingredient: temsirolimus, brand name: torisel drug)	Inhibition of mTORC1	Increase of autophagy flux	(Wang et al., 2011)
Beclin 1-derived peptide BCN4	BCN4 competes with Beclin 1 in binding to GAPR-1	Increase in the number of autophagosomes, autolysosomes, lysosomes	(Amin et al., 2024)
Nicotinamide mononucleotide (NMN)	Enhances acidification of acidic organelles	Increase of autophagy flux, reduction of the LC3-II/LC3-I ratio	(Liu et al., 2013)
Berberine	Activates AMPK and the class III PI3K	Increase of autophagy flux and lysosomal activity	(Zhuang et al., 2025)
Astragalin	Downregulated phosphorylation of PI3K/Akt-mTOR pathway-related proteins	Increase of autophagic flux and lysosomal activity	(Yang et al., 2023)
Resveratrol	AMPK-dependent inhibition of mTORC1	Increase in the number of autophagosomes	(Gong and Xia, 2020; Rahman et al., 2020)

In addition to synthetic drugs, natural compounds also exhibit neuroprotective potential by modulating autophagy. Certain natural compounds, including polyphenols, flavonoids, and alkaloids like resveratrol, astragalin, and berberine, have been shown to enhance autophagy. These compounds hold great potential, as several animal studies in mice demonstrated that they alleviate cognitive dysfunction associated with neurodegenerative diseases as well as toxin-induced neurotoxicity (Rahman et al., 2020; Tian et al., 2023; Yang et al., 2023).

Experimental and clinical use of pharmacologically available autophagy modulators should take into account their potential adverse effects which are summarized in Table 2. While some of the drugs have already been extensively screened for adverse effects (e.g., rapamycin, simvastatin, everolimus, and temsirolimus), adverse effects of other autophagic modulators are less well understood (Table 2).

**TABLE 2:** Possible side-effects of blood-brain barrier permeable autophagy activators.

Autophagy modulator	Molecular weight (g/mol)	Dosage	Adverse effects (AEs)	Tested organism	References
Rapamycin	914.2	15 ng/mL	Increase in infections, total cholesterol, LDL cholesterol and triglycerides in individuals with aging-related diseases in > 15 ng/mL in blood	Human	(de Wit et al., 2016; Lee et al., 2024)
Simvastatin	418.56	20 mg/day	Muscle pain, fatigue and weakness, rhabdomyolysis; AEs are dose dependent, risk is amplified by drug interactions that functionally increase statin potency	Human	(Golomb and Evans, 2008)
Everolimus	958.2	10 mg/day	Stomatitis, rash, fatigue, diarrhea, infections, nausea, appetite loss, hematologic toxicities, dyspnea, noninfectious pneumonitis, hypercholesterolemia, hyperglycemia	Human	(de Wit et al., 2016)
Temsirolimus	1030.3	25 mg/week	Anemia, hyperglycemia, asthenia, hypercholesterolemia, hypertriglyceridemia, hypophosphatemia	Human	(Bellmunt et al., 2008)
BCN4	1855.0	10 μg/day	Serum levels of ALT and immunoglobulin G (IgG) are unchanged	Mouse	(Amin et al., 2024)
Nicotinamide mononucleotide (NMN)	334.2	900 mg/day	No side effects (tested for 60 days)	Human	(Yi et al., 2023)
Berberine	336.4	90–3,000 mg	Constipation, nausea, diarrhea	Human	(Li et al., 2023)
Astragalin	448.4	25 μM/sample	Induction of apoptosis in human cancer cell lines a time- and concentration-dependent manner	Human cell lines	(You et al., 2017)
Resveratrol	228.2	2.5 g/day	Nausea, vomiting, diarrhea and liver dysfunction in patients with non-alcoholic fatty liver disease	Human	(Salehi et al., 2018)

Since astrocytes are key regulators of brain energy metabolism, autophagy modulation is of significant interest. When neuronal glucose supply is insufficient, especially under pathological conditions such as ischemic stroke, astrocytes support the survival and protection of neurons by providing metabolic substrates such as lactate and neurotropic factors (Yamagata, 2022). Nevertheless, the main source of energy for the human brain is glucose (Mergenthaler et al., 2013). Cells, especially astrocytes, dynamically detect its concentration to modulate their response to the availability of glucose. Glucose sensing is linked to mTOR activity within the mTORC1/mTORC2 complexes, which synchronizes the corresponding signaling pathways. Thus, under physiological conditions, when excess glucose is present, mTORC1 activates anabolic processes including protein, nucleotide, and lipid synthesis, which suppresses autophagy. On the other hand, mTORC1 is inhibited when glucose decreases, triggering autophagy, which helps maintain energy homeostasis and ensures cell survival and function, while limiting growth and proliferation. This is particularly important in neurodegenerative diseases that lead to disturbances in glucose-related metabolic pathways, including glycolysis, the pentose phosphate pathway, the tricarboxylic acid cycle and oxidative phosphorylation (McDonald et al., 2023). mTOR, however, is not the only autophagy regulator that senses energy status, as there are also other glucose sensors, such as AMPK. AMPK monitors cellular energy and initiates autophagy under low energy conditions by inhibiting mTORC1 (Leprivier and Rotblat, 2020). In addition to mTORC1, AMPK also modulates autophagy through interaction with the ULK1 complex. Under low glucose conditions, ULK1 is reportedly phosphorylated by AMPK, which then leads to the induction of autophagy (Kim et al., 2011), although this mechanism is still debated. Park and colleagues argue that AMPK controls the pace of autophagy by inhibiting its induction during energy deprivation to preserve essential autophagy components that are critical for maintaining cellular homeostasis and survival during energy stress (Park et al., 2023). An acute autophagy response to starvation corroborating this view, has already been observed in another study (Shang et al., 2011). Be that as it may, studies on AMPK knockout cells have clearly demonstrated that AMPK is not required for the activation of autophagy, as mTORC1 alone is sufficient (Kalender et al., 2010). In addition to glucose, lactate plays a central role in CNS metabolism, not only as a by-product of anaerobic metabolism, but also as an energy source. It is mainly produced by astrocytes from glucose or glycogen and serves as an important energy substrate for neurons and a signaling molecule (Suzuki et al., 2011). Lactate plays one of the central roles in metabolic regulation by bridging glycolysis and mitochondrial respiration, and regulates immunity, as described in tumors and viral infections (Mason, 2017). Recent studies have also shown that lactate drives lysine lactylation of histones and non-histone proteins to regulate gene expression and protein activity in numerous cell types, including macrophages, somatic cells, cancer and brain cells, in a glycolysis-dependent manner. Whether lactate regulates autophagy is still unknown. Cytosolic lactate levels are significantly increased under conditions of serum- or amino acid-deprivation. It is assumed that ULK1 phosphorylates LDHA in muscle and cancer cells under these conditions, thereby increasing LDHA activity and promoting the production of lactate. Regarding the modulation of autophagy, it is important to point out that lactate-mediated lactylation of the PI3K complex promotes its activity and activates autophagy, while also lactylating other key autophagy proteins such as ULK1 (Jia et al., 2023). The hypothesis that lactate-mediated lactylation of core autophagy proteins regulates autophagy activity in astrocytes and other cell types as outlined by Sun et al. (2023), remains to be investigated in astrocytes. While an increase in autophagy is beneficial, chronic overactivated autophagy may prove detrimental due to cytosolic accumulation of autophagosomes and increased degradation of essential cellular components (Ajoolabady et al., 2021).

## Conclusion

With neurological diseases being the leading cause of illness and disability worldwide, and many remaining untreatable, it is important to gain a better understanding of their underlying mechanisms. Research on neurological diseases has traditionally emphasized neurons, but emerging findings suggest that glial cells, may play a key role in the disrupted neuronal communication observed in various brain diseases. Reactive astrogliosis, a defining feature of astrocytes’ response to CNS pathologies, involves significant morphological, molecular and functional alterations, including dysregulated autophagy. By shaping metabolic and inflammatory responses, autophagy in astrocytes is without a doubt an important modulator of aging and neurodegeneration. The cytoskeletal network plays a critical role in astrocyte function under homeostatic and pathological conditions, particularly in reactive astrogliosis. Although autophagy is essential for the maintenance of neuron homeostasis in the brain, its regulation of autophagy in astrocytes, particularly its interaction with the cytoskeleton, remains poorly understood. Currently, it is not known how different types of cytoskeleton together with cytolinker proteins influence autophagy in astrocytes. However, it is known that IFs in astrocytes regulate the distribution and trafficking of vesicles, including lysosomes, proteolytic compartments with which autophagosomes fuse to degrade their cargo. All stages of autophagy, from the positioning and mobility of dysfunctional organelles, autophagosomes and lysosomes, are affected either directly or indirectly by the organization of the cytoskeleton. Direct effects are reflected in the intracellular positioning of lysosomes and autophagic compartments. The cytoskeleton affects the distribution of transporters at the plasma membrane, which enable the uptake of nutrients into astrocytes and the removal of metabolic waste into the glymphatic system, and thus indirectly influence the autophagy. Understanding how autophagy activity can be modulated in astrocytes is not only important to better understand its biological role in these cells but also for uncovering how astrocytic autophagy influences neuronal function and disfunction.

## References

[B1] Abbott N. J. Ronnback L. Hansson E. (2006). Astrocyte-endothelial interactions at the blood-brain barrier. *Nat. Rev. Neurosci.* 7 41–53. 10.1038/nrn1824 16371949

[B2] Aggarwal N. T. Mielke M. M. (2023). Sex differences in Alzheimer’s disease. *Neurol. Clin.* 41 343–358. 10.1016/j.ncl.2023.01.001 37030962 PMC10321561

[B3] Ajoolabady A. Wang S. Kroemer G. Penninger J. M. Uversky V. N. Pratico D. (2021). Targeting autophagy in ischemic stroke: From molecular mechanisms to clinical therapeutics. *Pharmacol. Ther.* 225:107848. 10.1016/j.pharmthera.2021.107848 33823204 PMC8263472

[B4] Amin A. Perera N. D. Tomas D. Cuic B. Radwan M. Hatters D. M. (2024). Systemic administration of a novel Beclin 1-derived peptide significantly upregulates autophagy in the spinal motor neurons of autophagy reporter mice. *Int. J. Pharm.* 659:124198. 10.1016/j.ijpharm.2024.124198 38816263

[B5] Baek A. Son S. Baek Y. M. Kim D. E. (2021). KRT8 (keratin 8) attenuates necrotic cell death by facilitating mitochondrial fission-mediated mitophagy through interaction with PLEC (plectin). *Autophagy* 17 3939–3956. 10.1080/15548627.2021.1897962 33783309 PMC8726610

[B6] Baldwin K. T. Murai K. K. Khakh B. S. (2023). Astrocyte morphology. *Trends Cell Biol.* 34 547–565. 10.1016/j.tcb.2023.09.006 38180380 PMC11590062

[B7] Ballou L. M. Lin R. Z. (2008). Rapamycin and mTOR kinase inhibitors. *J. Chem. Biol.* 1 27–36. 10.1007/s12154-008-0003-5 19568796 PMC2698317

[B8] Bellmunt J. Szczylik C. Feingold J. Strahs A. Berkenblit A. (2008). Temsirolimus safety profile and management of toxic effects in patients with advanced renal cell carcinoma and poor prognostic features. *Ann. Oncol.* 19 1387–1392. 10.1093/annonc/mdn066 18385198

[B9] Bhutia S. K. Mukhopadhyay S. Sinha N. Das D. N. Panda P. K. Patra S. K. (2013). Autophagy: Cancer’s friend or foe? *Adv. Cancer Res.* 118 61–95. 10.1016/B978-0-12-407173-5.00003-0 23768510 PMC4349374

[B10] Bishop G. M. Robinson S. R. (2004). Physiological roles of amyloid-beta and implications for its removal in Alzheimer’s disease. *Drugs Aging* 21 621–630. 10.2165/00002512-200421100-00001 15287821

[B11] Biskou O. Casanova V. Hooper K. M. Kemp S. Wright G. P. Satsangi J. (2019). The type III intermediate filament vimentin regulates organelle distribution and modulates autophagy. *PLoS One* 14:e0209665. 10.1371/journal.pone.0209665 30699149 PMC6353089

[B12] Borgione E. Lo Giudice M. Santa Paola S. Giuliano M. Lanza G. Cantone M. (2023). The Y831C mutation of the POLG gene in dementia. *Biomedicines* 11:1172. 10.3390/biomedicines11041172 37189790 PMC10136026

[B13] Caballero B. Bourdenx M. Luengo E. Diaz A. Sohn P. D. Chen X. (2021). Acetylated tau inhibits chaperone-mediated autophagy and promotes tau pathology propagation in mice. *Nat. Commun.* 12:2238. 10.1038/s41467-021-22501-9 33854069 PMC8047017

[B14] Carosi J. M. Sargeant T. J. (2019). Rapamycin and Alzheimer disease: A double-edged sword? *Autophagy* 15 1460–1462. 10.1080/15548627.2019.1615823 31066320 PMC6613906

[B15] Cassano T. Magini A. Giovagnoli S. Polchi A. Calcagnini S. Pace L. (2019). Early intrathecal infusion of everolimus restores cognitive function and mood in a murine model of Alzheimer’s disease. *Exp. Neurol.* 311 88–105. 10.1016/j.expneurol.2018.09.011 30243986

[B16] Chandrasekaran A. Dittlau K. S. Corsi G. I. Haukedal H. Doncheva N. T. Ramakrishna S. (2021). Astrocytic reactivity triggered by defective autophagy and metabolic failure causes neurotoxicity in frontotemporal dementia type 3. *Stem Cell Rep.* 16 2736–2751. 10.1016/j.stemcr.2021.09.013 34678206 PMC8581052

[B17] Crews L. Spencer B. Desplats P. Patrick C. Paulino A. Rockenstein E. (2010). Selective molecular alterations in the autophagy pathway in patients with Lewy body disease and in models of alpha-synucleinopathy. *PLoS One* 5:e9313. 10.1371/journal.pone.0009313 20174468 PMC2824828

[B18] De Strooper B. Karran E. (2016). The cellular phase of Alzheimer’s Disease. *Cell* 164 603–615. 10.1016/j.cell.2015.12.056 26871627

[B19] de Wit D. Schneider T. C. Moes D. J. Roozen C. F. den Hartigh J. Gelderblom H. (2016). Everolimus pharmacokinetics and its exposure-toxicity relationship in patients with thyroid cancer. *Cancer Chemother. Pharmacol.* 78 63–71. 10.1007/s00280-016-3050-6 27169792 PMC4921118

[B20] Deng Q. Wu C. Parker E. Liu T. C. Duan R. Yang L. (2024). Microglia and astrocytes in Alzheimer’s disease: Significance and summary of recent advances. *Aging Dis.* 15 1537–1564. 10.14336/AD.2023.0907 37815901 PMC11272214

[B21] Dhakal S. Subhan M. Fraser J. M. Gardiner K. Macreadie I. (2019). Simvastatin efficiently reduces levels of Alzheimer’s amyloid beta in yeast. *Int. J. Mol. Sci.* 20:3531. 10.3390/ijms20143531 31330953 PMC6678968

[B22] dos Santos G. Rogel M. R. Baker M. A. Troken J. R. Urich D. Morales-Nebreda L. (2015). Vimentin regulates activation of the NLRP3 inflammasome. *Nat. Commun.* 6:6574. 10.1038/ncomms7574 25762200 PMC4358756

[B23] Dragicevic N. Mamcarz M. Zhu Y. Buzzeo R. Tan J. Arendash G. W. (2010). Mitochondrial amyloid-beta levels are associated with the extent of mitochondrial dysfunction in different brain regions and the degree of cognitive impairment in Alzheimer’s transgenic mice. *J. Alzheimers Dis.* 20 (Suppl. 2), S535–S550. 10.3233/JAD-2010-100342 20463404

[B24] Escartin C. Galea E. Lakatos A. O’Callaghan J. P. Petzold G. C. Serrano-Pozo A. (2021). Reactive astrocyte nomenclature, definitions, and future directions. *Nat. Neurosci.* 24 312–325. 10.1038/s41593-020-00783-4 33589835 PMC8007081

[B25] Exner N. Lutz A. K. Haass C. Winklhofer K. F. (2012). Mitochondrial dysfunction in Parkinson’s disease: Molecular mechanisms and pathophysiological consequences. *EMBO J.* 31 3038–3062. 10.1038/emboj.2012.170 22735187 PMC3400019

[B26] Fang E. F. Hou Y. Palikaras K. Adriaanse B. A. Kerr J. S. Yang B. (2019). Mitophagy inhibits amyloid-beta and tau pathology and reverses cognitive deficits in models of Alzheimer’s disease. *Nat. Neurosci.* 22 401–412. 10.1038/s41593-018-0332-9 30742114 PMC6693625

[B27] Fass E. Shvets E. Degani I. Hirschberg K. Elazar Z. (2006). Microtubules support production of starvation-induced autophagosomes but not their targeting and fusion with lysosomes. *J. Biol. Chem.* 281 36303–36316. 10.1074/jbc.M607031200 16963441

[B28] Fassbender K. Simons M. Bergmann C. Stroick M. Lutjohann D. Keller P. (2001). Simvastatin strongly reduces levels of Alzheimer’s disease beta -amyloid peptides Abeta 42 and Abeta 40 in vitro and in vivo. *Proc. Natl. Acad. Sci. U. S. A.* 98 5856–5861. 10.1073/pnas.081620098 11296263 PMC33303

[B29] Feng Y. He D. Yao Z. Klionsky D. J. (2014). The machinery of macroautophagy. *Cell Res.* 24 24–41. 10.1038/cr.2013.168 24366339 PMC3879710

[B30] Filosa J. A. Morrison H. W. Iddings J. A. Du W. Kim K. J. (2016). Beyond neurovascular coupling, role of astrocytes in the regulation of vascular tone. *Neuroscience* 323 96–109. 10.1016/j.neuroscience.2015.03.064 25843438 PMC4592693

[B31] Frake R. A. Ricketts T. Menzies F. M. Rubinsztein D. C. (2015). Autophagy and neurodegeneration. *J. Clin. Invest.* 125 65–74. 10.1172/JCI73944 25654552 PMC4382230

[B32] Francois A. Rioux Bilan A. Quellard N. Fernandez B. Janet T. Chassaing D. (2014). Longitudinal follow-up of autophagy and inflammation in brain of APPswePS1dE9 transgenic mice. *J. Neuroinflamm.* 11:139. 10.1186/s12974-014-0139-x 25158693 PMC4154524

[B33] Frost G. R. Li Y. M. (2017). The role of astrocytes in amyloid production and Alzheimer’s disease. *Open Biol.* 7:170228. 10.1098/rsob.170228 29237809 PMC5746550

[B34] Futerman A. H. van Meer G. (2004). The cell biology of lysosomal storage disorders. *Nat. Rev. Mol. Cell Biol.* 5 554–565. 10.1038/nrm1423 15232573

[B35] Gabryel B. Kost A. Kasprowska D. Liber S. Machnik G. Wiaderkiewicz R. (2017). AMP-activated protein kinase is involved in induction of protective autophagy in astrocytes exposed to oxygen-glucose deprivation. *Cell Biol. Int.* 41 928–931. 10.1002/cbin.10793 28737298

[B36] Galluzzi L. Bravo-San Pedro J. M. Levine B. Green D. R. Kroemer G. (2017). Pharmacological modulation of autophagy: Therapeutic potential and persisting obstacles. *Nat. Rev. Drug Discov.* 16 487–511. 10.1038/nrd.2017.22 28529316 PMC5713640

[B37] Gan H. Ma Q. Hao W. Yang N. Chen Z. S. Deng L. (2024). Targeting autophagy to counteract neuroinflammation: A novel antidepressant strategy. *Pharmacol. Res.* 202:107112. 10.1016/j.phrs.2024.107112 38403256

[B38] Glover K. Li Y. Mukhopadhyay S. Leuthner Z. Chakravarthy S. Colbert C. L. (2017). Structural transitions in conserved, ordered Beclin 1 domains essential to regulating autophagy. *J. Biol. Chem.* 292 16235–16248. 10.1074/jbc.M117.804195 28798234 PMC5625053

[B39] Golomb B. A. Evans M. A. (2008). Statin adverse effects: A review of the literature and evidence for a mitochondrial mechanism. *Am. J. Cardiovasc. Drugs* 8 373–418. 10.2165/0129784-200808060-00004 19159124 PMC2849981

[B40] Gong C. Xia H. (2020). Resveratrol suppresses melanoma growth by promoting autophagy through inhibiting the PI3K/AKT/mTOR signaling pathway. *Exp. Ther. Med.* 19 1878–1886. 10.3892/etm.2019.8359 32104244 PMC7027143

[B41] Guo F. Liu X. Cai H. Le W. (2018). Autophagy in neurodegenerative diseases: Pathogenesis and therapy. *Brain Pathol.* 28 3–13. 10.1111/bpa.12545 28703923 PMC5739982

[B42] Hara T. Nakamura K. Matsui M. Yamamoto A. Nakahara Y. Suzuki-Migishima R. (2006). Suppression of basal autophagy in neural cells causes neurodegenerative disease in mice. *Nature* 441 885–889. 10.1038/nature04724 16625204

[B43] Hong B. Ohtake Y. Itokazu T. Yamashita T. (2024). Glial senescence enhances alpha-synuclein pathology owing to its insufficient clearance caused by autophagy dysfunction. *Cell Death Discov.* 10:50. 10.1038/s41420-024-01816-8 38272865 PMC10811334

[B44] Hung C. M. Lombardo P. S. Malik N. Brun S. N. Hellberg K. Van Nostrand J. L. (2021). AMPK/ULK1-mediated phosphorylation of Parkin ACT domain mediates an early step in mitophagy. *Sci. Adv.* 7:eabg4544. 10.1126/sciadv.abg4544 33827825 PMC8026119

[B45] Iliff J. J. Wang M. Liao Y. Plogg B. A. Peng W. Gundersen G. A. (2012). A paravascular pathway facilitates CSF flow through the brain parenchyma and the clearance of interstitial solutes, including amyloid beta. *Sci. Transl. Med.* 4:147ra111. 10.1126/scitranslmed.3003748 22896675 PMC3551275

[B46] Itoh N. Itoh Y. Meyer C. E. Suen T. T. Cortez-Delgado D. Rivera Lomeli M. (2023). Estrogen receptor beta in astrocytes modulates cognitive function in mid-age female mice. *Nat. Commun.* 14:6044. 10.1038/s41467-023-41723-7 37758709 PMC10533869

[B47] Iwaki H. Blauwendraat C. Leonard H. L. Makarious M. B. Kim J. J. Liu G. (2021). Differences in the presentation and progression of Parkinson’s disease by sex. *Mov. Disord.* 36 106–117. 10.1002/mds.28312 33002231 PMC7883324

[B48] Jia M. Yue X. Sun W. Zhou Q. Chang C. Gong W. (2023). ULK1-mediated metabolic reprogramming regulates Vps34 lipid kinase activity by its lactylation. *Sci. Adv.* 9:eadg4993. 10.1126/sciadv.adg4993 37267363 PMC10413652

[B49] Jia Q. Li S. Li X. J. Yin P. (2022). Neuroinflammation in Huntington’s disease: From animal models to clinical therapeutics. *Front. Immunol.* 13:1088124. 10.3389/fimmu.2022.1088124 36618375 PMC9815700

[B50] Jiang G. M. Tan Y. Wang H. Peng L. Chen H. T. Meng X. J. (2019). The relationship between autophagy and the immune system and its applications for tumor immunotherapy. *Mol. Cancer* 18:17. 10.1186/s12943-019-0944-z 30678689 PMC6345046

[B51] Jorgacevski J. Potokar M. (2023). Immune functions of astrocytes in viral neuroinfections. *Int. J. Mol. Sci.* 24:3514. 10.3390/ijms24043514 36834929 PMC9960577

[B52] Kadry H. Noorani B. Cucullo L. (2020). A blood-brain barrier overview on structure, function, impairment, and biomarkers of integrity. *Fluids Barriers CNS* 17:69. 10.1186/s12987-020-00230-3 33208141 PMC7672931

[B53] Kalender A. Selvaraj A. Kim S. Y. Gulati P. Brule S. Viollet B. (2010). Metformin, independent of AMPK, inhibits mTORC1 in a rag GTPase-dependent manner. *Cell Metab.* 11 390–401. 10.1016/j.cmet.2010.03.014 20444419 PMC3081779

[B54] Kast D. J. Dominguez R. (2017). The cytoskeleton-autophagy connection. *Curr. Biol.* 27 R318–R326. 10.1016/j.cub.2017.02.061 28441569 PMC5444402

[B55] Kim J. Kundu M. Viollet B. Guan K. L. (2011). AMPK and mTOR regulate autophagy through direct phosphorylation of Ulk1. *Nat. Cell Biol.* 13 132–141. 10.1038/ncb2152 21258367 PMC3987946

[B56] Kim S. Chun H. Kim Y. Park U. Chu J. Bhalla M. (2024). Astrocytic autophagy plasticity modulates Aβ clearance and cognitive function in Alzheimer’s disease. *Mol. Neurodegener.* 19:55. 10.1186/s13024-024-00740-w 39044253 PMC11267931

[B57] Kochl R. Hu X. W. Chan E. Y. Tooze S. A. (2006). Microtubules facilitate autophagosome formation and fusion of autophagosomes with endosomes. *Traffic* 7 129–145. 10.1111/j.1600-0854.2005.00368.x 16420522

[B58] Lee D. J. W. Hodzic Kuerec A. Maier A. B. (2024). Targeting ageing with rapamycin and its derivatives in humans: A systematic review. *Lancet Healthy Longev.* 5 e152–e162. 10.1016/S2666-7568(23)00258-1 38310895

[B59] Leng F. Edison P. (2021). Neuroinflammation and microglial activation in Alzheimer disease: Where do we go from here? *Nat. Rev. Neurol.* 17 157–172. 10.1038/s41582-020-00435-y 33318676

[B60] Leng K. Rooney B. McCarthy F. Xia W. Rose I. V. L. Bax S. (2024). mTOR activation induces endolysosomal remodeling and nonclassical secretion of IL-32 via exosomes in inflammatory reactive astrocytes. *J. Neuroinflamm.* 21:198. 10.1186/s12974-024-03165-w 39118084 PMC11312292

[B61] Leprivier G. Rotblat B. (2020). How does mTOR sense glucose starvation? AMPK is the usual suspect. *Cell Death Discov.* 6:27. 10.1038/s41420-020-0260-9 32351714 PMC7176732

[B62] Li X. Alafuzoff I. Soininen H. Winblad B. Pei J. J. (2005). Levels of mTOR and its downstream targets 4E-BP1, eEF2, and eEF2 kinase in relationships with tau in Alzheimer’s disease brain. *FEBS J.* 272 4211–4220. 10.1111/j.1742-4658.2005.04833.x 16098202

[B63] Li Z. Wang Y. Xu Q. Ma J. Li X. Yan J. (2023). Berberine and health outcomes: An umbrella review. *Phytother. Res.* 37 2051–2066. 10.1002/ptr.7806 36999891

[B64] Liu D. Pitta M. Jiang H. Lee J. H. Zhang G. Chen X. (2013). Nicotinamide forestalls pathology and cognitive decline in Alzheimer mice: Evidence for improved neuronal bioenergetics and autophagy procession. *Neurobiol. Aging* 34 1564–1580. 10.1016/j.neurobiolaging.2012.11.020 23273573 PMC3596471

[B65] Liu J. Liu W. Li R. Yang H. (2019). Mitophagy in Parkinson’s Disease: From pathogenesis to treatment. *Cells* 8:712. 10.3390/cells8070712 31336937 PMC6678174

[B66] Liu T. Wang P. Yin H. Wang X. Lv J. Yuan J. (2023). Rapamycin reverses ferroptosis by increasing autophagy in MPTP/MPP(+)-induced models of Parkinson’s disease. *Neural Regen. Res.* 18 2514–2519. 10.4103/1673-5374.371381 37282484 PMC10360095

[B67] Liu X. Tian F. Wang S. Wang F. Xiong L. (2018). Astrocyte autophagy flux protects neurons against oxygen-glucose deprivation and ischemic/reperfusion injury. *Rejuvenation Res.* 21 405–415. 10.1089/rej.2017.1999 29125039

[B68] Lo C. H. Zeng J. (2023). Defective lysosomal acidification: A new prognostic marker and therapeutic target for neurodegenerative diseases. *Transl. Neurodegener.* 12:29. 10.1186/s40035-023-00362-0 37287072 PMC10249214

[B69] Ma R. Y. Li L. Yang H. Zou B. Ma R. X. Zhang Y. (2024). Therapeutic effect of nicotinamide mononucleotide on Alzheimer’s disease through activating autophagy and anti-oxidative stress. *Biomed. Pharmacother.* 178:117199. 10.1016/j.biopha.2024.117199 39053426

[B70] Marder K. S. Tang M. X. Mejia-Santana H. Rosado L. Louis E. D. Comella C. L. (2010). Predictors of parkin mutations in early-onset Parkinson disease: The consortium on risk for early-onset Parkinson disease study. *Arch. Neurol.* 67 731–738. 10.1001/archneurol.2010.95 20558392 PMC3329757

[B71] Martinez-Vicente M. (2017). Neuronal mitophagy in neurodegenerative diseases. *Front. Mol. Neurosci.* 10:64. 10.3389/fnmol.2017.00064 28337125 PMC5340781

[B72] Mason S. (2017). Lactate shuttles in neuroenergetics-homeostasis. Allostasis and Beyond. *Front. Neurosci.* 11:43. 10.3389/fnins.2017.00043 28210209 PMC5288365

[B73] Matsumoto G. Wada K. Okuno M. Kurosawa M. Nukina N. (2011). Serine 403 phosphorylation of p62/SQSTM1 regulates selective autophagic clearance of ubiquitinated proteins. *Mol. Cell* 44 279–289. 10.1016/j.molcel.2011.07.039 22017874

[B74] Matthews P. M. (2019). Chronic inflammation in multiple sclerosis - seeing what was always there. *Nat. Rev. Neurol.* 15 582–593. 10.1038/s41582-019-0240-y 31420598

[B75] Mayne K. White J. A. McMurran C. E. Rivera F. J. de la Fuente A. G. (2020). Aging and neurodegenerative disease: Is the adaptive immune system a friend or foe? *Front. Aging Neurosci.* 12:572090. 10.3389/fnagi.2020.572090 33173502 PMC7538701

[B76] McConnell H. L. Kersch C. N. Woltjer R. L. Neuwelt E. A. (2017). The translational significance of the neurovascular unit. *J. Biol. Chem.* 292 762–770. 10.1074/jbc.R116.760215 27920202 PMC5247651

[B77] McDonald T. S. Lerskiatiphanich T. Woodruff T. M. McCombe P. A. Lee J. D. (2023). Potential mechanisms to modify impaired glucose metabolism in neurodegenerative disorders. *J. Cereb. Blood Flow Metab.* 43 26–43. 10.1177/0271678X221135061 36281012 PMC9875350

[B78] McKnight N. C. Zhenyu Y. (2013). Beclin 1, an essential component and master regulator of PI3K-III in health and disease. *Curr. Pathobiol. Rep.* 1 231–238. 10.1007/s40139-013-0028-5 24729948 PMC3979578

[B79] Mergenthaler P. Lindauer U. Dienel G. A. Meisel A. (2013). Sugar for the brain: The role of glucose in physiological and pathological brain function. *Trends Neurosci.* 36 587–597. 10.1016/j.tins.2013.07.001 23968694 PMC3900881

[B80] Monastyrska I. Rieter E. Klionsky D. J. Reggiori F. (2009). Multiple roles of the cytoskeleton in autophagy. *Biol. Rev. Camb. Philos. Soc.* 84 431–448. 10.1111/j.1469-185X.2009.00082.x 19659885 PMC2831541

[B81] Natale G. Limanaqi F. Busceti C. L. Mastroiacovo F. Nicoletti F. Puglisi-Allegra S. (2021). Glymphatic system as a gateway to connect neurodegeneration from periphery to CNS. *Front. Neurosci.* 15:639140. 10.3389/fnins.2021.639140 33633540 PMC7900543

[B82] Nguyen T. N. Padman B. S. Lazarou M. (2016). Deciphering the molecular signals of PINK1/Parkin mitophagy. *Trends Cell Biol.* 26 733–744. 10.1016/j.tcb.2016.05.008 27291334

[B83] Osipova E. D. Semyachkina-Glushkovskaya O. V. Morgun A. V. Pisareva N. V. Malinovskaya N. A. Boitsova E. B. (2018). Gliotransmitters and cytokines in the control of blood-brain barrier permeability. *Rev. Neurosci.* 29 567–591. 10.1515/revneuro-2017-0092 29306934

[B84] Palmer J. E. Wilson N. Son S. M. Obrocki P. Wrobel L. Rob M. (2025). Autophagy, aging, and age-related neurodegeneration. *Neuron* 113 29–48. 10.1016/j.neuron.2024.09.015 39406236

[B85] Pan P. Su L. Wang X. Chai W. Liu D. Song L. (2021). Vimentin regulation of autophagy activation in lung fibroblasts in response to lipopolysaccharide exposure in vitro. *Ann. Transl. Med.* 9:304. 10.21037/atm-20-5129 33708931 PMC7944268

[B86] Pardridge W. M. (2005). The blood-brain barrier: Bottleneck in brain drug development. *NeuroRx* 2 3–14. 10.1602/neurorx.2.1.3 15717053 PMC539316

[B87] Park J. M. Lee D. H. Kim D. H. (2023). Redefining the role of AMPK in autophagy and the energy stress response. *Nat. Commun.* 14:2994. 10.1038/s41467-023-38401-z 37225695 PMC10209092

[B88] Perez-Alvarez M. J. Villa Gonzalez M. Benito-Cuesta I. Wandosell F. G. (2018). Role of mTORC1 controlling proteostasis after brain ischemia. *Front. Neurosci.* 12:60. 10.3389/fnins.2018.00060 29497356 PMC5818460

[B89] Picca A. Faitg J. Auwerx J. Ferrucci L. D’Amico D. (2023). Mitophagy in human health, ageing and disease. *Nat. Metab.* 5 2047–2061. 10.1038/s42255-023-00930-8 38036770 PMC12159423

[B90] Potokar M. Jorgacevski J. (2021). Plectin in the central nervous system and a putative role in brain astrocytes. *Cells* 10:2353. 10.3390/cells10092353 34572001 PMC8464768

[B91] Potokar M. Morita M. Wiche G. Jorgacevski J. (2020). The diversity of intermediate filaments in astrocytes. *Cells* 9:1604. 10.3390/cells9071604 32630739 PMC7408014

[B92] Potokar M. Stenovec M. Gabrijel M. Li L. Kreft M. Grilc S. (2010). Intermediate filaments attenuate stimulation-dependent mobility of endosomes/lysosomes in astrocytes. *Glia* 58 1208–1219. 10.1002/glia.21000 20544856

[B93] Potokar M. Stenovec M. Kreft M. Kreft M. E. Zorec R. (2008). Stimulation inhibits the mobility of recycling peptidergic vesicles in astrocytes. *Glia* 56 135–144. 10.1002/glia.20597 17990309

[B94] Price B. R. Johnson L. A. Norris C. M. (2021). Reactive astrocytes: The nexus of pathological and clinical hallmarks of Alzheimer’s disease. *Ageing Res. Rev.* 68:101335. 10.1016/j.arr.2021.101335 33812051 PMC8168445

[B95] Rahman M. H. Akter R. Bhattacharya T. Abdel-Daim M. M. Alkahtani S. Arafah M. W. (2020). Resveratrol and neuroprotection: Impact and its therapeutic potential in Alzheimer’s disease. *Front. Pharmacol.* 11:619024. 10.3389/fphar.2020.619024 33456444 PMC7804889

[B96] Ravikumar B. Sarkar S. Rubinsztein D. C. (2008). Clearance of mutant aggregate-prone proteins by autophagy. *Methods Mol. Biol.* 445 195–211. 10.1007/978-1-59745-157-4_13 18425452

[B97] Ravikumar B. Vacher C. Berger Z. Davies J. E. Luo S. Oroz L. G. (2004). Inhibition of mTOR induces autophagy and reduces toxicity of polyglutamine expansions in fly and mouse models of Huntington disease. *Nat. Genet.* 36 585–595. 10.1038/ng1362 15146184

[B98] Romano R. Cordella P. Bucci C. (2025). The type III intermediate filament protein peripherin regulates lysosomal degradation activity and autophagy. *Int. J. Mol. Sci.* 26:549. 10.3390/ijms26020549 39859265 PMC11766092

[B99] Salehi B. Mishra A. P. Nigam M. Sener B. Kilic M. Sharifi-Rad M. (2018). Resveratrol: A double-edged sword in health benefits. *Biomedicines* 6:91. 10.3390/biomedicines6030091 30205595 PMC6164842

[B100] Shang L. Chen S. Du F. Li S. Zhao L. Wang X. (2011). Nutrient starvation elicits an acute autophagic response mediated by Ulk1 dephosphorylation and its subsequent dissociation from AMPK. *Proc. Natl. Acad. Sci. U. S. A.* 108 4788–4793. 10.1073/pnas.1100844108 21383122 PMC3064373

[B101] Son S. Baek A. Lee J. H. Kim D. E. (2022). Autophagosome-lysosome fusion is facilitated by plectin-stabilized actin and keratin 8 during macroautophagic process. *Cell Mol. Life Sci.* 79:95. 10.1007/s00018-022-04144-1 35080691 PMC11072119

[B102] Sun Q. Xu X. Wang T. Xu Z. Lu X. Li X. (2021). Neurovascular units and neural-glia networks in intracerebral hemorrhage: From mechanisms to translation. *Transl. Stroke Res.* 12 447–460. 10.1007/s12975-021-00897-2 33629275

[B103] Sun W. Jia M. Feng Y. Cheng X. (2023). Lactate is a bridge linking glycolysis and autophagy through lactylation. *Autophagy* 19 3240–3241. 10.1080/15548627.2023.2246356 37565742 PMC10621282

[B104] Suzuki A. Stern S. A. Bozdagi O. Huntley G. W. Walker R. H. Magistretti P. J. (2011). Astrocyte-neuron lactate transport is required for long-term memory formation. *Cell* 144 810–823. 10.1016/j.cell.2011.02.018 21376239 PMC3073831

[B105] Tajes M. Ramos-Fernandez E. Weng-Jiang X. Bosch-Morato M. Guivernau B. Eraso-Pichot A. (2014). The blood-brain barrier: Structure, function and therapeutic approaches to cross it. *Mol. Membr. Biol.* 31 152–167. 10.3109/09687688.2014.937468 25046533

[B106] Tansey M. G. Wallings R. L. Houser M. C. Herrick M. K. Keating C. E. Joers V. (2022). Inflammation and immune dysfunction in Parkinson disease. *Nat. Rev. Immunol.* 22 657–673. 10.1038/s41577-022-00684-6 35246670 PMC8895080

[B107] Tavcar Verdev P. Potokar M. Korva M. Resman Rus K. Kolenc M. Avsic Zupanc T. (2022). In human astrocytes neurotropic flaviviruses increase autophagy, yet their replication is autophagy-independent. *Cell Mol. Life Sci.* 79:566. 10.1007/s00018-022-04578-7 36283999 PMC9596533

[B108] Tian E. Sharma G. Dai C. (2023). Neuroprotective properties of berberine: Molecular mechanisms and clinical implications. *Antioxidants* 12:1883. 10.3390/antiox12101883 37891961 PMC10604532

[B109] Tsunemi T. Ishiguro Y. Yoroisaka A. Valdez C. Miyamoto K. Ishikawa K. (2020). Astrocytes protect human dopaminergic neurons from alpha-synuclein accumulation and propagation. *J. Neurosci.* 40 8618–8628. 10.1523/JNEUROSCI.0954-20.2020 33046546 PMC7643299

[B110] Vaillant-Beuchot L. Mary A. Pardossi-Piquard R. Bourgeois A. Lauritzen I. Eysert F. (2021). Accumulation of amyloid precursor protein C-terminal fragments triggers mitochondrial structure, function, and mitophagy defects in Alzheimer’s disease models and human brains. *Acta Neuropathol.* 141 39–65. 10.1007/s00401-020-02234-7 33079262 PMC7785558

[B111] Valencia R. G. Mihailovska E. Winter L. Bauer K. Fischer I. Walko G. (2021). Plectin dysfunction in neurons leads to tau accumulation on microtubules affecting neuritogenesis, organelle trafficking, pain sensitivity and memory. *Neuropathol. Appl. Neurobiol.* 47 73–95. 10.1111/nan.12635 32484610 PMC7891324

[B112] Verkhratsky A. Nedergaard M. (2018). Physiology of Astroglia. *Physiol. Rev.* 98 239–389. 10.1152/physrev.00042.2016 29351512 PMC6050349

[B113] Verkhratsky A. Matteoli M. Parpura V. Mothet J. P. Zorec R. (2016). Astrocytes as secretory cells of the central nervous system: Idiosyncrasies of vesicular secretion. *EMBO J.* 35 239–257. 10.15252/embj.201592705 26758544 PMC4741299

[B114] Viscomi M. T. D’Amelio M. Cavallucci V. Latini L. Bisicchia E. Nazio F. (2012). Stimulation of autophagy by rapamycin protects neurons from remote degeneration after acute focal brain damage. *Autophagy* 8 222–235. 10.4161/auto.8.2.18599 22248716

[B115] Wang S. Long H. Hou L. Feng B. Ma Z. Wu Y. (2023). The mitophagy pathway and its implications in human diseases. *Signal Transduct. Target Ther.* 8:304. 10.1038/s41392-023-01503-7 37582956 PMC10427715

[B116] Wang X. Jia J. (2023). Magnolol improves Alzheimer’s disease-like pathologies and cognitive decline by promoting autophagy through activation of the AMPK/mTOR/ULK1 pathway. *Biomed. Pharmacother.* 161:114473. 10.1016/j.biopha.2023.114473 36889111

[B117] Wang Y. Wang X. Y. Subjeck J. R. Shrikant P. A. Kim H. L. (2011). Temsirolimus, an mTOR inhibitor, enhances anti-tumour effects of heat shock protein cancer vaccines. *Br. J. Cancer* 104 643–652. 10.1038/bjc.2011.15 21285988 PMC3049595

[B118] Webb J. L. Ravikumar B. Rubinsztein D. C. (2004). Microtubule disruption inhibits autophagosome-lysosome fusion: Implications for studying the roles of aggresomes in polyglutamine diseases. *Int. J. Biochem. Cell Biol.* 36 2541–2550. 10.1016/j.biocel.2004.02.003 15325591

[B119] Wiche G. Winter L. (2011). Plectin isoforms as organizers of intermediate filament cytoarchitecture. *Bioarchitecture* 1 14–20. 10.4161/bioa.1.1.14630 21866256 PMC3158638

[B120] Wilkins H. M. (2023). Interactions between amyloid, amyloid precursor protein, and mitochondria. *Biochem. Soc. Trans.* 51 173–182. 10.1042/BST20220518 36688439 PMC9987971

[B121] Winter L. Kuznetsov A. V. Grimm M. Zeöld A. Fischer I. Wiche G. (2015). Plectin isoform P1b and P1d deficiencies differentially affect mitochondrial morphology and function in skeletal muscle. *Hum. Mol. Genet.* 24 4530–4544. 10.1093/hmg/ddv184 26019234 PMC4512624

[B122] Xiang J. Liu X. Ren J. Chen K. Wang H. L. Miao Y. Y. (2019). How does estrogen work on autophagy? *Autophagy* 15 197–211. 10.1080/15548627.2018.1520549 30208759 PMC6333457

[B123] Yamagata K. (2022). Lactate supply from astrocytes to neurons and its role in ischemic stroke-induced neurodegeneration. *Neuroscience* 481 219–231. 10.1016/j.neuroscience.2021.11.035 34843897

[B124] Yang C. Z. Wang S. H. Zhang R. H. Lin J. H. Tian Y. H. Yang Y. Q. (2023). Neuroprotective effect of astragalin via activating PI3K/Akt-mTOR-mediated autophagy on APP/PS1 mice. *Cell Death Discov.* 9:15. 10.1038/s41420-023-01324-1 36681681 PMC9867706

[B125] Yang Q. Q. Zhou J. W. (2019). Neuroinflammation in the central nervous system: Symphony of glial cells. *Glia* 67 1017–1035. 10.1002/glia.23571 30548343

[B126] Yazdankhah M. Ghosh S. Liu H. Hose S. Zigler J. S. Jr. Sinha D. (2023). Mitophagy in astrocytes is required for the health of optic nerve. *Cells* 12:2496. 10.3390/cells12202496 37887340 PMC10605486

[B127] Yi L. Maier A. B. Tao R. Lin Z. Vaidya A. Pendse S. (2023). The efficacy and safety of beta-nicotinamide mononucleotide (NMN) supplementation in healthy middle-aged adults: A randomized, multicenter, double-blind, placebo-controlled, parallel-group, dose-dependent clinical trial. *Geroscience* 45 29–43. 10.1007/s11357-022-00705-1 36482258 PMC9735188

[B128] You O. H. Shin E. A. Lee H. Kim J. H. Sim D. Y. Kim J. H. (2017). Apoptotic effect of astragalin in melanoma skin cancers via activation of caspases and inhibition of sry-related HMg-box gene 10. *Phytother. Res.* 31 1614–1620. 10.1002/ptr.5895 28809055

[B129] Zhang W. Xiao D. Mao Q. Xia H. (2023). Role of neuroinflammation in neurodegeneration development. *Signal Transduct. Target Ther.* 8:267. 10.1038/s41392-023-01486-5 37433768 PMC10336149

[B130] Zhuang W. Huang Z. Yu L. Yu M. He H. Deng Y. (2025). Berberine enhances autophagic flux to alleviate ischemic neuronal injury by facilitating N-ethylmaleimide-sensitive factor-mediated fusion of autophagosomes with lysosomes. *Biochem. Pharmacol.* 232:116715. 10.1016/j.bcp.2024.116715 39672277

[B131] Zimmermann A. Madeo F. Diwan A. Sadoshima J. Sedej S. Kroemer G. (2024). Metabolic control of mitophagy. *Eur. J. Clin. Invest.* 54:e14138. 10.1111/eci.14138 38041247

[B132] Zugec M. Furlani B. Castanon M. J. Rituper B. Fischer I. Broggi G. (2024). Plectin plays a role in the migration and volume regulation of astrocytes: A potential biomarker of glioblastoma. *J. Biomed. Sci.* 31:14. 10.1186/s12929-024-01002-z 38263015 PMC10807171

[B133] Zureick A. H. McFadden K. A. Mody R. Koschmann C. (2019). Successful treatment of a TSC2-mutant glioblastoma with everolimus. *BMJ Case Rep.* 12:e227734. 10.1136/bcr-2018-227734 31154346 PMC6557420

